# Preliminary insights into intratesticular and intraepididymal administration of eugenol: histological alterations and impact on sperm motility in Wistar rats

**DOI:** 10.1590/1984-3143-AR2025-0026

**Published:** 2026-03-20

**Authors:** Renner Philipe Rodrigues Carvalho, Iara Magalhaes Ribeiro, Camilo Ramirez-Lopez, Tayná Bolsam da Silva, Alex Filipe Ramos de Sousa, Arabela Guedes de Azevedo Viana, Victor de Medeiros, Ana Carolina Marta Trindade, Thanyanne Rafaela de Morais Ferreira, Luiz Otávio Guimaraes Ervilha, Mariana Machado-Neves

**Affiliations:** 1 Laboratório de Biologia Estrutural, Departamento de Biologia Geral, Universidade Federal de Viçosa, Viçosa, MG, Brasil; 2 Programa de Pós-graduação em Medicina Veterinária, Departamento de Veterinária, Universidade Federal de Viçosa, Viçosa, MG, Brasil

**Keywords:** chemical castration, Syzygium aromaticum, toxicology, morphology

## Abstract

This study assessed the histopathological and functional consequences of single bilateral intratesticular (InT; 200, 20, and 10 mg) and intraepididymal (InE; 50, 5, and 2.5 mg) eugenol administration in *Wistar* rats. Animals were monitored for up to 60 days, and testicular and epididymal tissues were examined for morphological alterations and sperm motility impairment. Both administration routes induced marked structural damage to the male reproductive tract. InT application led to progressive degeneration of the seminiferous epithelium, tubular atrophy, interstitial fibrosis, and focal mineralization, accompanied by reductions in both Leydig cell volumetric proportion and nuclear volume, hallmarks of impaired spermatogenesis and disrupted lumicrine signaling. In contrast, InE administration caused epithelial necrosis, intraluminal cellular debris accumulation, fibrotic remodeling, and ductal occlusion, ultimately leading to complete loss of sperm motility due to impaired maturation and obstructed transport. Although no overt systemic toxicity was observed, the findings indicate that local eugenol delivery exerts direct cytotoxic effects and promotes irreversible tissue remodeling. These results highlight eugenol’s potential as a chemical sterilant and reinforce the need for further studies to evaluate the reversibility of reproductive damage, inflammatory sequelae, and interspecies variability in toxicological responses.

## Introduction

Male reproductive health has garnered significant attention due to its pivotal role in human fertility (Khourdaji et al., 2018), wildlife conservation (Holt and Comizzoli, 2022), and livestock productivity (Thundathil et al., 2016). Moreover, the pursuit of novel male contraceptive approaches, both reversible and permanent, has intensified, encompassing not only pharmacological modulation but also chemical sterilization strategies as alternatives to surgical castration (Khourdaji et al., 2018; Hess et al., 2024). In parallel, the pharmacological exploitation of natural products has gained considerable traction, particularly regarding phytochemicals with the potential to interfere with spermatogenesis, steroidogenesis, and epididymis function (Carvalho et al., 2022). Several plant-derived compounds have been reported to impair male fertility through endocrine disruption, germ cell apoptosis, or impairment of sperm maturation (Abshenas et al., 2013).

Among these phytochemicals, clove oil (*Syzygium aromaticum*) has attracted particular attention for its potential application in chemical sterilization protocols, owing to its high content of eugenol, a bioactive phenolic compound with potent pharmacological properties. Experimental studies in dogs (Abu-Ahmed, 2015; Aponte et al., 2024) and pigs (Trindade et al., 2023) have shown that InT administration of clove oil promotes pronounced testicular degeneration, suppression of steroidogenesis, and, in some cases, irreversible infertility. Nonetheless, these effects are markedly influenced by interspecies variability, dosage, and administration route. Eugenol (4-allyl-2-methoxyphenol; C_10_H_12_O_2_), the predominant phenolic constituent of clove oil, exhibits a dual pharmacological profile: at low concentrations, it confers cytoprotective effects via antioxidant and anti-inflammatory pathways, whereas at higher doses, it exhibits cytotoxic effects, functioning as a pro-oxidant and inducing oxidative stress, mitochondrial dysfunction, and apoptosis in various tissues (Aburel et al., 2021; Carvalho et al., 2022, Carvalho et al., 2025).

While the systemic pharmacodynamics of eugenol have been extensively characterized, its direct impact on male reproductive structures, particularly the testes and epididymides, remains insufficiently elucidated. Mechanistically, eugenol’s high lipophilicity enables membrane partitioning and mitochondrial dysfunction (complex I inhibition/ΔΨm dissipation), with dose-dependent redox effec ts and modulation of inflammatory and cell-cycle pathways. However, how these processes affect spermatogenesis and epididymis function remains poorly characterized and not fully elucidated (e.g., Aburel et al., 2021; Taghipour et al., 2023). Given the epididymis’s pivotal role in sperm maturation, acquisition of motility, and post-testicular sperm viability (Robaire and Hinton, 2015; Dacheux and Dacheux, 2014), the local effects of eugenol on this organ deserve further investigation. In this context, the objective of this study was to evaluate the histological alterations and effects on sperm motility resulting from intratesticular (InT) and intraepididymal (InE) administration of eugenol in Wistar rats at 15, 30, and 60 days post-injection.

## Methods

**Figure 1:**
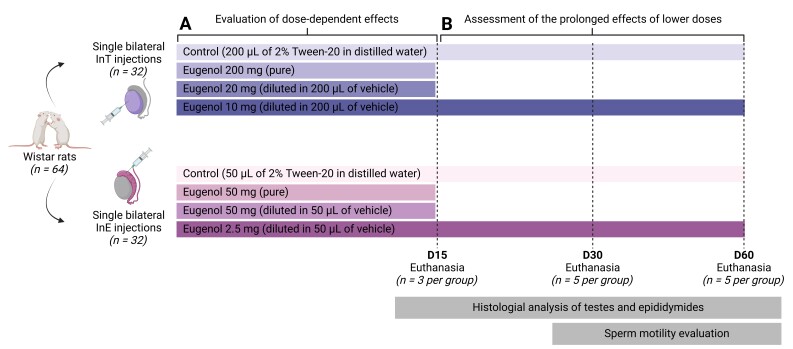
Experimental design for intratesticular (InT) and intraepididymal (InE) eugenol administration in *Wistar* rats. A total of 64 *Wistar* rats were divided into two experimental phases. (A) In the first phase, 24 rats (*n = 3 per group*) were randomly assigned to evaluate the dose-dependent effects of eugenol. Animals received a single bilateral injection of either the vehicle (2% Tween-20 in distilled water) or different doses of eugenol in the testes (InT) or epididymal caput (InE). InT groups included: (1) Control (200 μL of the vehicle), (2) Eugenol 200 mg (pure), (3) Eugenol 20 mg (diluted in 200 μL of the vehicle), and (4) Eugenol 10 mg (diluted in 200 μL of the vehicle). InE groups included: (5) Control (50 μL of the vehicle), (6) Eugenol 50 mg (pure), (7) Eugenol 5 mg (diluted in 50 μL of the vehicle), and (8) Eugenol 2.5 mg (diluted in 50 μL of the vehicle). Euthanasia was performed on day 15 (D15), and testes and epididymides were collected for histological analysis. (B) In the second phase, 40 rats (*n = 10 per group*) were used to assess the prolonged effects of the lowest eugenol doses. The experimental groups were: (1) Control (InT; 200 μL of the vehicle), (2) Eugenol 10 mg (InT; diluted in 200 μL of the vehicle), (3) Control (InE; 50 μL of the vehicle), and (4) Eugenol 2.5 mg (InE; diluted in 50 μL of the vehicle). Animals were euthanized at D30 and D60 (*n = 5 per timepoint*), and tissues were collected for histological analysis and sperm motility evaluation.

Sixty-four *Wistar* rats (70 days old; 230–250 g) were obtained from the Central Animal Facility of the Universidade Federal de Viçosa (UFV) and housed individually in polypropylene cages under controlled photoperiod (12 h light/12 h dark) and temperature (21 ± 2 °C). Food and water were provided ad libitum. The experimental design and procedures were approved by the Ethics Committee on Animal Use of UFV (CEUA/UFV, protocol no. 35/2023) and conducted in accordance with the guidelines of the Brazilian National Council for the Control of Animal Experimentation (CONCEA). Throughout the study, animals were monitored daily for signs of distress, pain, or behavioral alterations, including changes in locomotion, feeding, and grooming behavior. The study was divided into two phases (Figure 1). In phase one, 24 rats were randomly assigned to eight groups (n = 3/group) to evaluate the effects of different doses of eugenol (Sigma-Aldrich) (Figure 1A). Four groups received bilateral InT injections: (1) Control: 200 μL of 2% Tween-20 in distilled water (used as vehicle); (2) Eugenol 200 mg (pure); (3) Eugenol 20 mg; and (4) Eugenol 10 mg, with the latter two diluted in 200 μL of vehicle. The remaining four groups received bilateral InE injections in the caput epididymis: (5) Control: 50 μL of vehicle; (6) Eugenol 50 mg (pure); (7) Eugenol 5 mg; and (8) Eugenol 2.5 mg, with the latter two diluted in 50 μL of vehicle. All injections were performed on day 0 (D0) following sedation with ketamine (75 mg kg^−1^) and xylazine (10 mg kg^−1^). A scrotal approach was used in all animals. For the InT group, the needle was inserted through the scrotal skin at the caudal pole and gently advanced into the middle third of the testicular parenchyma, avoiding the rete testis and cauda epididymis. For the InE group, injections were directed into the caput epididymides, which is readily palpable and well defined in Wistar rats. In both cases, solutions were delivered slowly using insulin syringes (29G; 0.33 mm × 13 mm) to minimize tissue trauma and reflux. Animals were monitored daily, and euthanasia was performed on day 15 (D15) via deep anesthesia with xylazine (45 mg Kg^-1^) and ketamine hydrochloride (240 mg Kg^-1^). Testes and epididymides were collected and fixed in Bouin's solution for 24 hours.

Phase two focused on the prolonged effects of the lowest eugenol doses. Forty rats were divided into four groups (n = 10/group; Figure 1B). Two groups received InT injections: (1) Control: 200 μL of vehicle; (2) Eugenol 10 mg diluted in 200 μL of vehicle. The other two groups received InE injections: (3) Control: 50 μL of vehicle; (4) Eugenol 2.5 mg diluted in 50 μL of vehicle. Injections were administered on D0, and animals were monitored daily. Five animals per group were euthanized on days 30 (D30) and 60 (D60) for tissue collection. Testes and epididymides were fixed in Bouin's solution for 24 hours, and cauda epididymis samples were collected for sperm motility evaluation.

Testicular and epididymal tissues were dehydrated in ethanol, embedded in 2-hydroxyethyl methacrylate (Historesin®, Leica), sectioned at 3 μm using a rotary microtome (RM 2255, Leica), stained with hematoxylin and eosin, and mounted with Entellan (Merck). Qualitative analyses were performed under a light microscope at 10x, 20x, and 40x magnifications (Carvalho et al., 2022). Digital images of testicular parenchyma were acquired using a photomicroscope (Olympus BX-53, Tokyo, Japan) and analyzed using Image-Pro Plus 4.5 software (Media Cybernetics, Silver Spring, MD, USA). The volumetric proportion (%) of Leydig cells was obtained by counting 1,000 points projected onto ten images per animal from the intertubular compartment. Moreover, 50 nuclei of Leydig cells were chosen randomly in either circular or elliptical form, and their volume was measured using image analysis software Leica Q-win (version 3) with the aid of the software ImageJ 1.48. The nuclear volume of Leydig cells was determined by the mathematical formula: [Diameter^3^× π × 1/6] (Barros et al., 2018). These morphometric analyses were performed on testicular samples from animals subjected to InT eugenol administration and evaluated at 30 and 60 days.

For sperm motility evaluation, fragments from the cauda epididymis were incubated in 1 mL of Biggers-Whitten-Whittingham medium at 37°C for 5 min. Aliquots of the supernatant were placed between a pre-warmed slide and a coverslip. Sperm motility was assessed by analyzing 200 spermatozoa per animal under a phase-contrast microscope (L-1000B, Bioval) at 400x magnification, classifying them as motile or immotile (Carvalho et al., 2022). Statistical analysis was performed exclusively on sperm motility and Leydig cell morphometric data. The Shapiro–Wilk test was used to assess the normality of the data distribution. Comparisons between control and eugenol-injected groups were conducted using *Student’s T-test*. Differences were considered statistically significant at p < 0.05. All analyses and graphical representations were performed using GraphPad Prism 6.0 (GraphPad Software Inc., San Diego, CA, USA). Data are expressed as mean ± standard deviation (SD).

## Results and discussion

This study offers novel insights into the histopathological and functional repercussions of single-dose bilateral administration of eugenol via InT and InE routes in *Wistar* rats. Both approaches elicited pronounced alterations in the structural integrity of testicular and epididymal tissues, resulting in the disruption of spermatogenesis and/or impairment of sperm maturation. The observed degenerative and necrotic changes within the seminiferous epithelium and epididymal epithelium underscore the direct cytotoxic potential of eugenol upon contact with male reproductive structures. Notably, the complete absence of sperm motility in animals receiving InE injections strongly suggests epididymal duct obstruction or collapse, ultimately precluding sperm maturation and transport. Throughout the observation period, none of the eugenol-injected animals exhibited overt clinical signs of discomfort, behavioral changes, gastrointestinal distress, or systemic toxicity. Nevertheless, the possibility of subclinical or delayed onset of adverse effects, such as chronic inflammation or immune-mediated responses, warrants further investigation. Collectively, these findings reinforce eugenol’s reproductive toxicity and inform ongoing efforts to develop a chemical sterilant with defined safety profiles for potential application in fertility control protocols.

**Figure 2:**
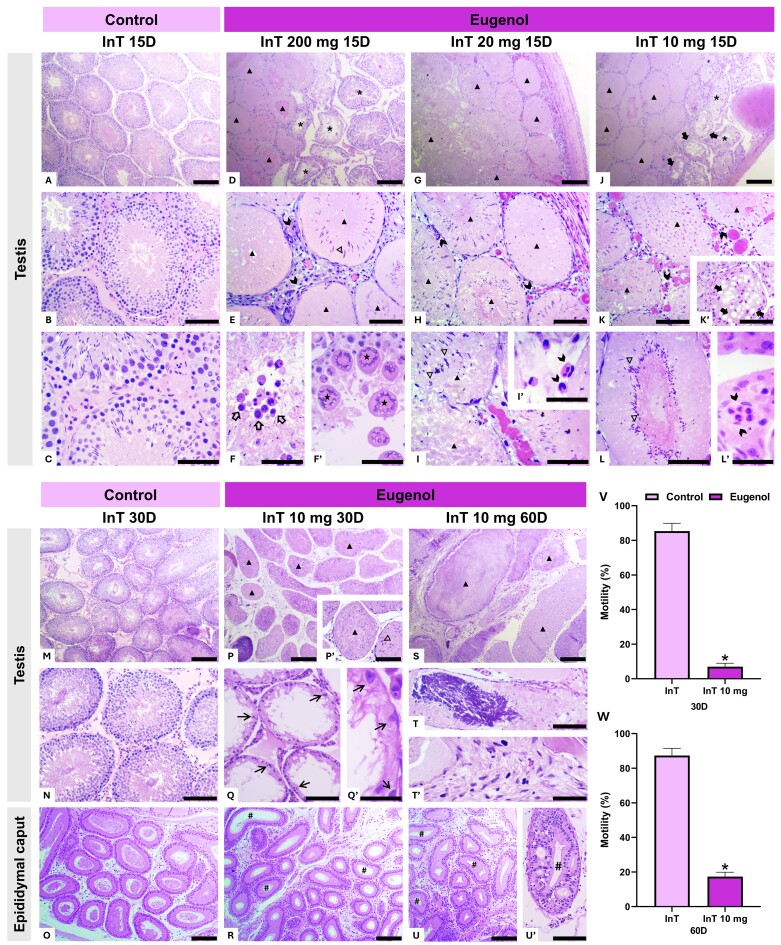
Histological sections of testis and caput epididymis and sperm motility of *Wistar* rats, at 15, 30, and 60 days (D) after single bilateral intratesticular (InT) administration of different doses of eugenol (200, 20, and 10 mg). A, B, and C show the typical histological architecture of the control group 15 days after the InT administration of the vehicle, characterized by seminiferous tubules with an organized seminiferous epithelium, germ cells at different stages of development, and spermatozoa in the lumen, as well as a preserved intertubular compartment with Leydig cells and blood vessels. D, E, F, and F’ show seminiferous tubules undergoing degeneration (*) and partial necrosis (▲), polymorphonuclear leukocytes in the intertubular compartment (➤), residual spermatozoa in the seminiferous tubules (△), exfoliation of germ cells (⇨), and multinucleated giant cells in the seminiferous tubules (★) at 15 days after the InT administration of 200 mg of eugenol. G, H, I, and I’ show necrotic seminiferous tubules (▲), polymorphonuclear leukocytes in the intertubular compartment (➤), and residual spermatozoa in the seminiferous tubules (△) at 15 days after the InT administration of 20 mg of eugenol. J, K, K’, L, and L’ show necrotic seminiferous tubules (▲), seminiferous tubules undergoing degeneration and atrophy (*), vacuolization of seminiferous tubules (⮕), increased congestion of blood vessels, polymorphonuclear leukocytes in the intertubular compartment (➤), and residual spermatozoa in the seminiferous tubules (△) at 15 days after the InT administration of 10 mg of eugenol. M and N show the typical histological architecture of the control group after 30 days of InT vehicle administration, with the preserved organization of the seminiferous tubules and intertubular compartment. In O, the typical histological architecture of the caput epididymis is observed, characterized by a pseudostratified columnar epithelium, with spermatozoa in the lumen and preserved connective stroma. P, P’, Q, and Q’ show necrotic seminiferous tubules (▲), residual spermatozoa in the seminiferous tubules (△), and seminiferous tubules completely devoid of germ cells lined only by Sertoli cells (→). In R, a complete absence of spermatozoa in the lumen of the caput epididymis ducts (#) is observed 30 days after the InT administration of 10 mg of eugenol. In S, necrotic seminiferous tubules (▲) are observed; in T, a fine granular to amorphous laminated basophilic material and possible mineralization are noted, and in T’, the presence of fibers indicates fibrosis. In U and U’, a complete absence of spermatozoa in the lumen of the caput epididymis (#) is observed, as well as epithelial vacuolization at 60 days after the InT administration of 10 mg of eugenol. Scale bars: 100 μm. Sperm motility percentages of *Wistar* rats at 30 and 60 days after InT eugenol administration (10 mg) are shown in graphs V and W, respectively. Significant difference (p < 0.05) between control and eugenol-injected groups by *Student’s t-test*. Control group: 2% Tween-20 (60 days); Eugenol groups: eugenol diluted in 2% Tween-20. (n = 5 animals/group).

Histological examination of the testes and caput epididymis showed significant time-dependent morphological changes following InT eugenol administration. At 15 days post-injection, control animals that received vehicle application alone exhibited well-organized seminiferous tubules with a structured epithelium, germ cells at various stages of development, abundant luminal spermatozoa, and a preserved intertubular compartment containing Leydig cells and blood vessels (Figure 2A, 2B, 2C). Conversely, animals that received InT injections of eugenol exhibited pronounced histopathological alterations. In the 200 mg and 20 mg groups, seminiferous tubules showed extensive degeneration and necrosis, polymorphonuclear leukocyte infiltration, the presence of residual spermatozoa, exfoliation of germ cells, and the formation of multinucleated giant cells (Figure 2D, 2E, 2F, 2F’, 2G, 2H, 2I, 2I’). Even at the lowest dose (10 mg), InT administration of eugenol induced significant architectural disruption, marked by necrosis, degeneration, atrophy, vacuolization, increased vascular congestion, and inflammatory infiltration. At the same time, some seminiferous tubules still harbored resilient spermatozoa (Figure 2J, 2K, 2K’, 2L, 2L’). In a few tubules, some cells compatible with undifferentiated spermatogonia were still distinguishable near the basement membrane, although they appeared reduced in number compared with controls.

These testicular histopathological alterations align with previously reported toxic effects of eugenol. Although eugenol is generally considered safe as a food additive (Carvalho et al., 2022), its cytotoxic potential increases when administered directly into tissues (Aburel et al., 2021). Upon direct application, eugenol may act similarly to sclerosing agents (Oliveira et al., 2012; Paranzini et al., 2018), compromising the blood-testis barrier and potentially triggering an autoimmune response, where anti-sperm antibodies are generated, further exacerbating tissue damage. Additionally, due to its lipophilic nature, eugenol may increase cell membrane permeability, disrupt ionic homeostasis, and induce oxidative stress through reactive oxygen species production (Carvalho et al., 2022). These effects are comparable to the cytotoxic mechanisms observed in zinc oxide-eugenol materials, widely used in dentistry. These have been reported to induce chronic inflammation, degeneration, and necrosis, particularly when in direct contact with vital tissues (Nejad et al., 2017). Despite extensive testicular necrosis, spermatozoa were still detected in some tubules, suggesting that sperm produced before the injury had not been fully degraded, likely due to the structural stability conferred by their highly condensed chromatin (Wu and Chu, 2008). These findings indicate that eugenol-induced toxicity extends beyond direct chemical disruption, potentially initiating a systemic immunological response that perpetuates tissue damage over time.

At 30 days post-InT injection, control animals maintained preserved testicular architecture, with intact seminiferous tubules and an undisturbed intertubular compartment (Figure 2M, 2N). The caput epididymis exhibited a pseudostratified columnar epithelium, with spermatozoa in the lumen and preserved connective stroma (Figure 2O). Conversely, animals that received 10 mg of eugenol via InT displayed severe histopathological damage, characterized by necrotic seminiferous tubules, the presence of residual spermatozoa, and complete depletion of germ cells, with some tubules still retaining a few Sertoli cells lining the basement membrane (Figure 2P, 2P’, 2Q, 2Q’). These structural findings were corroborated by the morphometric analysis of the intertubular compartment, which revealed a significant reduction in Leydig cell volumetric proportion, as well as decreases in nuclear and cytoplasmic fractions and smaller nuclear diameters when compared with control animals (p < 0.05; Table 1). At this point, only the lowest dose was used, as earlier observations indicated that InT application of 10 mg eugenol induced testicular damage comparable to higher doses.

**Table 1:** Morphometric parameters of Leydig cells of Wistar rats at 30 and 60 days after intratesticular (InT) administration of eugenol (10 mg).

	Leydig cell volumetric proportion (%)	Nuclear proportion (%)	Cytoplasmic proportion (%)	Nuclear diameter (μm)
*30 days*				
InT Control	9.52 ± 0.78	1.74 ± 0.22	7.78 ± 0.54	165.48 ± 11.85
InT Eugenol 10 mg	3.82 ± 0.46^*^	0.72 ± 0.10*	3.10 ± 0.38*	133.54 ± 10.7*
*60 days*				
InT Control	8.04 ± 0.67	1.60 ± 0.20	6.44 ± 0.42	158.62 ± 13.88
InT Eugenol 10 mg	2.14 ± 0.31*	0.40 ± 0.07*	1.74 ± 0.24*	128.36 ± 11.72*

Mean ± SD. Control group: Rats receiving vehicle (2% Tween-20) by intratesticular injection; Eugenol groups: Rats receiving eugenol (10 mg; diluted in 2% Tween-20) by intratesticular injection. Asterisks (*) indicate a significant difference (p < 0.05) between control and eugenol-treated groups by Student’s t-test. (n = 5 rats/group).

By 60 days, while the testicular and epididymal architecture remained intact in control animals (data not shown), InT-administered eugenol resulted in further tissue deterioration, with seminiferous tubules largely disorganized and only rarely showing any cells morphologically compatible with germ or Sertoli cells. Besides extensive necrosis, laminated basophilic material suggestive of dystrophic mineralization (Foster, 2016) was present in the testes, along with fibrotic fibers (Figure 2S, 2T, 2T’). These findings indicate progressive tissue injury, characterized by fibrosis, epithelial vacuolization, and mineralization, which are reparative processes that fail to restore standard functionality. Fibrosis typically replaces necrotic testicular parenchyma with fibroblasts and collagen deposition (Xu et al., 2024). At the same time, vacuole formation likely represents spaces left by lost germ cells during germinal atrophy (Cesta et al., 2014). Additionally, mineralization, frequently associated with sperm stasis, was observed in the capsule, blood vessels, and seminiferous tubules, further compromising tissue architecture (Cesta et al., 2014). Consistent with these histological alterations, morphometric evaluation also revealed a significant reduction in Leydig cell volumetric proportion, as well as decreased nuclear and cytoplasmic fractions and smaller nuclear diameters compared with controls (p < 0.05; Table 1), reinforcing the progressive impairment of the testicular damage at this later time point.

**Figure 3:**
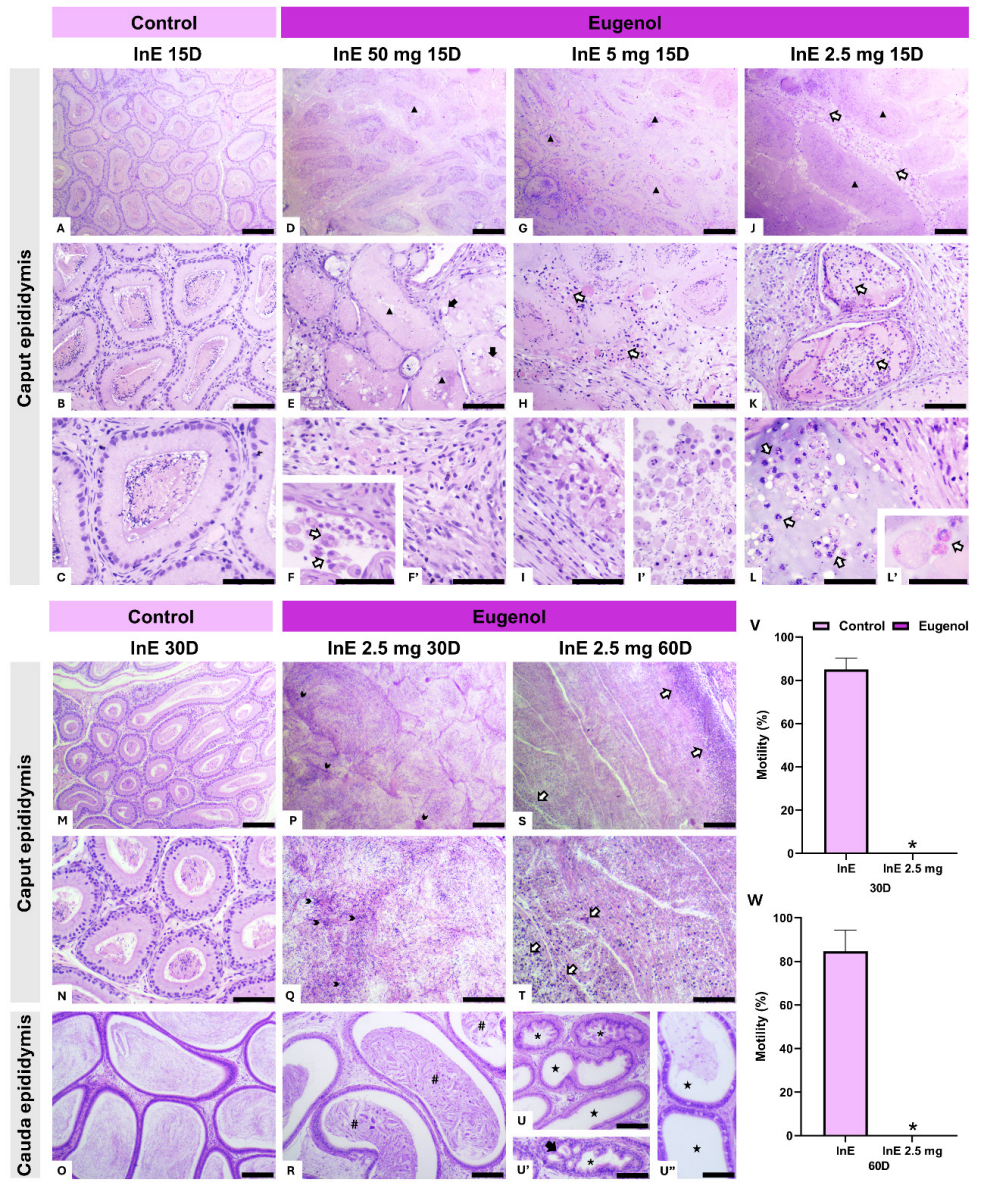
Histological sections of the epididymis and sperm motility of *Wistar* rats at 15, 30, and 60 days (D) after a single bilateral intraepididymal (InE) administration of different doses of eugenol (50, 5, and 2.5 mg). A, B, and C show the typical histological architecture of the caput epididymis in the control group 15 days after InE administration of the vehicle, characterized by a pseudostratified columnar epithelium, the presence of spermatozoa in the lumen, and a preserved connective stroma. D and E show widespread degeneration and necrosis with structural deterioration of the ducts (▲) and epithelial vacuolization (⮕). F and F’ show polymorphonuclear leukocytes in the duct lumen (⇨), while in F’, an increase in blood vessels and connective fibers, characteristic of fibrosis, is observed 15 days after the InE administration of 50 mg of eugenol. G and H show necrotic epididymal ducts (▲) and polymorphonuclear leukocytes in the interductal compartment (⇨). I and I’ show massive infiltration of immune cells and cellular debris in the duct lumen 15 days after the InE administration of 5 mg of eugenol. J, K, L, and L’ show necrotic epididymal ducts (▲) and polymorphonuclear leukocytes both in the interductal compartment and within the epididymal ducts (⇨) 15 days after the InE administration of 2.5 mg of eugenol. M, N, and O show the typical histological architecture of the control group after 30 days of InE vehicle administration, with a preserved organization of the caput and cauda epididymis, characterized by a pseudostratified columnar epithelium, the presence of spermatozoa in the lumen, and a preserved connective stroma. P and Q show generalized ductal necrosis with loss of tubular structure, but clusters of residual spermatozoa (➤) are still observed. In R, the sections of the cauda epididymis exhibit an intact epithelium, and the lumen contains cellular debris and fibrous-like material (#) 30 days after the InE administration of 2.5 mg of eugenol. S and T demonstrate fibrous deposition in the caput epididymis, consistent with fibrosis, as well as dispersed and aggregated polymorphonuclear leukocytes (⇨). In U, U’, and U”, sections of the cauda epididymis show ducts devoid of spermatozoa (★), a cribriform appearance (*), and epithelial vacuolization (⮕) at 60 days after the InE administration of 2.5 mg of eugenol. Scale bars: 100 μm. Sperm motility percentages of *Wistar* rats at 30 and 60 days after InE eugenol administration (2.5 mg) are shown in graphs V and W, respectively. Significant difference (p < 0.05) between control and eugenol-injected groups by *Student’s t-test*. Control group: 2% Tween-20 (60 days); Eugenol groups: eugenol diluted in 2% Tween-20. (n = 5 animals/group).

At 15 days post-InE administration, a similar pattern of tissue damage was observed as in the InT injections, reinforcing the toxic effects of eugenol on male reproductive structures (Abu-Ahmed, 2015; Trindade et al., 2023; Aponte et al., 2024). Control animals displayed a normal caput epididymis, with well-differentiated ducts lined by a pseudostratified columnar epithelium, abundant luminal spermatozoa, and a preserved connective stroma (Figure 3A, 3B, 3C). In contrast, animals that received InE injections exhibited acute structural damage. Ductal necrosis, epithelial vacuolization, and leukocyte infiltration were prominent in all groups, with increasing severity at higher doses (Figure 3D, 3E, 3F, 3F’, 3G, 3H, 3I, 3I’, 3J). In several regions, pronounced immune cell infiltration and accumulation of cellular debris within the ductal lumen were evident, indicating a robust inflammatory response (Cesta et al., 2014; Figure 3K, 3L, 3L’). Similar findings were described by Leoci et al. (2019), who reported necrotic epididymal sections surrounded by dense collagen fiber encapsulation and calcium deposits following InE injection of calcium chloride in dogs. The fibrotic response and encapsulation observed in the surrounding connective tissue in our study (Figure 3K, 3L) suggest ongoing tissue remodeling, consistent with the chronic inflammatory and necrotizing action of sclerosing agents. As observed in InT injections, the lowest InE dose (2.5 mg) induced comparable damage to the higher doses, leading to its selection for evaluation at 30 and 60 days.

At 30 days post-InE administration, control animals maintained a well-organized epididymal architecture, with a pseudostratified epithelium, preserved lumen filled with spermatozoa, and intact connective stroma (Figure 3M, 3N, 3O). Conversely, in animals receiving InE eugenol injections, tissue damage became more extensive, with generalized ductal necrosis and loss of tubular structure. Clusters of residual spermatozoa were still observable in some regions (Figure 3P, 3Q). Still, the lumen contained significant amounts of cellular debris and fibrous-like material in many areas, particularly in the cauda epididymis (Kempinas and Klinefelter, 2015; Figure 3R). This accumulation of debris suggests that the local inflammatory response and tissue degradation may have contributed to ductal obstruction, impeding sperm transport (Azenabor et al., 2015; Colpi and Caroppo, 2024).

By 60 days post-InE administration, while the caput and cauda architecture remained intact in control animals (data not shown), the extent of caput epididymis damage had increased significantly, with widespread fibrotic deposition, persistent leukocyte infiltration, and severe structural disruption (Figure 3S, 3T). Although spermatozoa could still be retrieved from the cauda epididymis, they exhibited complete immotility, reinforcing that epididymis dysfunction rather than sperm absence was responsible for the reproductive impairment. This observation supports the well-established role of the epididymis in the acquisition of motility and fertilizing ability, which depends on proper epithelial function, luminal fluid composition, and intercellular signaling (Robaire and Hinton, 2015; Dacheux and Dacheux, 2014). Structural or functional disruption of the epididymal epithelium can impair sperm maturation, leading to the release of immotile or non-functional sperm despite their presence (Gatti et al., 2004; Figure 3U, 3U’, 3U”). Given the progressive fibrotic remodeling observed, it is plausible that these spermatozoa originated from spermatogenesis prior to the InE injection, remaining stored in the cauda epididymis, as previously demonstrated in ligated rabbits, where spermatozoa persisted for weeks without numerical loss despite degeneration (Jones, 2004). At the same time, new sperm production was prevented from reaching the epididymal lumen due to ductal obstruction. These findings align with reports that epididymis obstruction, often a consequence of inflammation or fibrotic remodeling, can disrupt sperm transport and contribute to post-testicular infertility by blocking the excurrent duct system, leading to azoospermia or severe oligozoospermia (Jequier, 2011; Azenabor et al., 2015; Colpi and Caroppo, 2024). Functionally, this severe structural alteration corresponded with a total loss of sperm motility (Figure 3W), a stark contrast to the residual motility observed following InT administration. Given the critical role of the epididymis in sperm maturation, these findings indicate that InE administration not only interferes with sperm transport but also compromises the epididymal microenvironment, which is extremely necessary for sperm viability (Dacheux and Dacheux, 2014; Robaire and Hinton, 2015; Zhou et al., 2019). Further long-term studies are warranted to determine whether chronic inflammatory responses or sperm granuloma formation may occur in the caput epididymis following chemical insult, potentially leading to persistent tissue remodeling and reproductive complications. Previous studies have demonstrated that disruption of the epididymal epithelium, particularly under conditions of testosterone depletion, can lead to extravasation of spermatozoa and round spermatids into the interstitium, triggering granulomatous inflammation, immune cell infiltration, and local fibrosis (Dutta et al., 2019). These pathological processes are especially relevant in the context of non-surgical sterilization strategies targeting the epididymis, and their potential long-term consequences should be carefully evaluated.

While the findings of this study highlight eugenol’s potential as a chemical sterilant, important considerations regarding its long-term safety and reversibility must be addressed. One critical concern is whether the histopathological damage observed in the testes and epididymides is permanent or reversible over extended periods. The extensive fibrosis and mineralization detected in the testicular parenchyma, along with epididymal ductal necrosis and obstruction, suggest that tissue remodeling might lead to irreversible infertility (Leoci et al., 2019). Potential systemic inflammatory responses cannot be ruled out, as eugenol-induced tissue damage could trigger prolonged immune activation, with implications for overall reproductive and endocrine function (Carvalho et al., 2022). Another key aspect to consider is species-specific variability in response to eugenol exposure. Although the results obtained in *Wistar* rats provide valuable insights, differences in reproductive physiology across species (Hess et al., 2024) necessitate further studies to evaluate the safety and efficacy of eugenol in larger animal models, particularly those used in livestock production or as contraceptive targets in wildlife management. Addressing these uncertainties will be crucial for determining whether eugenol-based formulations could be safely and effectively implemented in long-term reproductive control strategies.

## Conclusion

In summary, both InT and InE administration of eugenol severely damaged male reproductive structures through distinct mechanisms. InT administration disrupted testicular architecture, causing seminiferous tubule degeneration and altering key morphometric parameters of Leydig cells, which likely compromised androgen synthesis and contributed to the observed decline in sperm motility. InE administration targeted the epididymis, inducing ductal necrosis, epithelial vacuolization, and sperm transport obstruction, resulting in complete loss of motile sperm in the cauda. Taken together, these outcomes indicate that eugenol acts directly on both germ cells and somatic components, particularly Sertoli, Leydig, and epididymal epithelial cells, promoting local cytotoxicity, structural destabilization, and secondary inflammatory–fibrotic remodeling. These findings support the feasibility of eugenol as a sterilization agent, though concerns remain regarding irreversibility, systemic inflammation, and species-specific variability. Because this study focused on morphological and functional endpoints, the precise molecular pathways and cell-specific targets remain unresolved.
